# Protective effects of butein on corticosterone-induced cytotoxicity in Neuro2A cells

**DOI:** 10.1016/j.ibror.2020.02.002

**Published:** 2020-03-03

**Authors:** Masanori Ohmoto, Yukina Shibuya, Shihori Taniguchi, Tomoki Nakade, Masaaki Nomura, Yuri Ikeda-Matsuo, Tohru Daikoku

**Affiliations:** a Department of Pharmacy Practice and Sciences, Faculty of Pharmaceutical Sciences, Hokuriku University, Japan; b Department of Clinical Pharmacy, Faculty of Pharmaceutical Sciences, Hokuriku University, Japan; c Department of Pharmaceutical Life Sciences, Faculty of Pharmaceutical Sciences, Hokuriku University, Japan

**Keywords:** Butein, Corticosterone, Apoptosis, Neurite outgrowth, ROS, Neuro2A cells

## Abstract

A functional understanding of the relationship between glucocorticoids and neuronal apoptosis induced by the production of reactive oxygen species (ROS) may lead to a novel strategy for the treatment or prevention of depression. Previous reports suggest that butein, a type of flavonoids, may be a potent candidate against depression-related neuronal cell apoptosis caused by oxidative stress; however, the protective effects of butein on damaged corticosterone (CORT)-treated neuronal cells has not been elucidated. In the present study, we examined the protective effect of butein on CORT-induced cytotoxicity and neurite growth during cell differentiation of mouse neuroblastoma Neuro2A (N2A) cells. Moreover, the effect on cultured cells by high concentrations of butein was confirmed. Our results demonstrate that CORT treatment significantly decreases cell viability and induces cell death. CORT was suggested to induce apoptosis via mitochondrial dysfunction and caspase-3 activation; this apoptosis may be attributed to DNA damage by ROS generation, found in this study to be significantly inhibited by pretreatment with butein. We found that CORT produced significant growth suppression of retinoic acid-induced neurite outgrowth in N2A cells; however, butein significantly increased neurite length and induced dose-dependent apoptotic cytotoxicity in N2A cells. This study suggests that low concentration of butein can prevent CORT-induced cytotoxicity in N2A cells, and provides preliminary results supporting some of the beneficial roles of butein in neuroprotection.

## Highlights

• Butein protected Neuro2A cells from CORT-induced apoptosis via mitochondrial dysfunction, caspase-3 activation, and DNA damage.
• CORT suppressed retinoic acid-induced neurite outgrowth in Neuro2A cells.
• Butein inhibited CORT-suppressed neurite outgrowth in Neuro2A cells.
• High doses of butein induced cytotoxicity in Neuro2A cells.

## Introduction

Prolonged glucocorticoid (GC) elevation due to chronic stress has been shown to reduce hippocampal volume and impair hippocampus-dependent functions, leading to the development of depression (Nestler et al., 2002; Manji et al., 2001; Gould et al., 1998; Lupien et al., 1998). Plasma GC levels are constantly controlled through a negative feedback mechanism in HPA axis regulation. The pathophysiology of depression is hypothesized to result in GC overexposure of the brain and hippocampus by HPA axis dysregulation. Because the hippocampus is particularly sensitive to GCs, excessive GC levels causes hippocampal damage and cell loss. Neuronal cells in the brain are particularly vulnerable to oxidative stress. Past reports have suggested that GC elevation generates reactive oxygen species (ROS) and causes damage to neuronal cells (e.g. in the hippocampus), inducing cell death via apoptosis (Sousa et al., 1999; Sato et al., 2010; Latt et al., 2018). Elucidating the functional understanding of the relationship between glucocorticoids and neuronal apoptosis focused on ROS production may pave a novel strategy for the treatment/prevention of depression.

Liu et al. (2011) and Zhang et al. (2012) demonstrated the protective effects of icariin as a type of flavonoid on corticosterone (CORT)-induced apoptosis in several cultured neurons, and found that icariin inhibits intracellular accumulation of ROS in CORT-treated cells. Flavonoids possess a basic phenyl-benzopyrone core and include chalcones, flavanones, and their derivatives. Many flavonoids possess inhibitory effects against oxidative stress and ROS production in relation to antidepressant activities (Guan and Liu, 2016; Hritcu et al., 2017). The chalcones of our interest are found in many fruits and vegetables; they possess a wide range of beneficial pharmacological effects. Butein, one of these chalcones, has exhibited potential therapeutic and protective effects as demonstrated in various models of human chronic diseases (Song et al., 2016; Padmavathi et al., 2017). The research groups of Guan and Liu showed that butein had the most potential as an anti-depressive and had significantly diminished immobility time in the mouse forced swim test compared with other synthesized chalcone derivatives (Guan and Liu, 2016). Cho et al. (2012) demonstrated that butein significantly protected glutamate-induced neurotoxicity, attenuated ROS production, and suppressed the expression of both inducible nitric oxide synthase and cyclooxygenase-2 by stimulation with a lipopolysaccharide in a dose-dependent manner. Similar potent inhibitory activities have been reported with regards to the effect of butein on glutamate neurotoxicity in cortical cells of primary cultured rats (Cho et al., 2013). These reports suggest that butein could be a potent candidate against a depression derived from neuronal cell apoptosis by oxidative stress. Despite this potential, the protective effects of butein on damaged CORT-treated neuronal cells have yet to be elucidated.

In the present study, we examined the protective effects of butein against CORT-induced cytotoxicity using mouse neuroblastoma Neuro2A (N2A) cells, a cell line used frequently to study neuroprotective abilities of various factors (Ghaffari et al., 2014; Furukawa et al., 2019) and the neurotoxicity of putative drugs (Shastry et al., 2001). The N2A cells are also extensively used in studies concerning neuronal differentiation and neurite growth (Matta et al., 1986), in which their cellular and molecular mechanisms (Radio and Mundy, 2008) have been elucidated. It is well known that N2A cells differentiate into a neuronal-like phenotype and elaborate neurites upon serum reduction and retinoic acid treatment, (Bulfone et al., 2005). This well-defined neuronal model is often implemented in studies relating to neuronal differentiation. It is also a popular cell line for studying neurotoxicity, as the brain is the first target in conditions such as neurodegeneration and neuronal dysfunction. Neurite outgrowth is the differentiation process by which neurons establish synapses. In the dentate gyrus of the hippocampus, new neurons are constantly produced and undergo neurite outgrowth to form synapses. This injury is closely related to depression, which is manifested by hippocampal nerve regeneration disorder and neurotrophic and synaptic plasticity deficits. A recent study (Meng et al., 2019) indicated that leonurine prevents CORT-induced neurotoxicity and consequent damaged cell morphology in PC12 cells by reducing neurite outgrowth. However, it remains unclear whether butein prevents negative effects of CORT on neurite outgrowth. Thus, we evaluated the effect of butein in CORT-induced cell disturbance and neurite outgrowth in N2A cells. Moreover, the effects of high concentrations of butein on cultured cells were confirmed.

## Materials and methods

### Cell culture

N2A cells were cultured in E-MEM (Fujifilm Wako, Japan) supplemented with 10 % fetal bovine serum (FBS) and penicillin/streptomycin (Fujifilm Wako, Japan), in a humidified atmosphere of 5 % CO_2_ at 37 °C.

### Treatment preparation

Butein (Tokyo Chemical Industry, Japan) and CORT (Fujifilm Wako, Japan) were dissolved in dimethyl sulfoxide (DMSO) (Fujifilm Wako, Japan) and the ﬁnal concentration of DMSO was made up to less than 0.01 % for all treatments. It was assumed that the effect of DMSO was negligible and control cultures were treated in medium without DMSO.

### Cell viability and cytotoxicity

An MTT assay was used to measure cell viability and lactate dehydrogenase (LDH) release was used to assess cell death. Cells were seeded into 96-well plates (5 × 10^4^ cells/mL) and incubated overnight at 37℃. After exposure to butein and CORT, cells were treated with MTT (Dojindo Molecular Technologies, Japan) dissolved in PBS (final concentration of 0.5 mg/mL per well) and incubated at 37 °C for 2.5 h. The supernatant was removed, followed by the addition of DMSO to dissolve the precipitate. The absorbance of MTT formazan was measured at a wavelength of 535 nm. The percent cytotoxicity was estimated using the LDH assay kit (Dojindo Molecular Technologies, Japan). The absorbance in MTT and LDH assays were detected using a microplate reader (Tecan, Switzerland). To assess the concentration-dependent cytotoxicity of butein, trypan blue staining was performed. Cell suspensions were added in equal parts of 0.4 % trypan blue dye (Fujifilm Wako, Japan). Cells were counted using the Neubauer improved cell counting chambers. The percentage of trypan blue-positive cells (dead cells) against the number of total cells was calculated to evaluate the degree of cell death.

### DNA damage detection assay

To assess DNA damage, an assay was performed using the phosphorylated histone H2AX (γH2AX) marker for double strand breaks. Cells were seeded (2 × 10^4^ cells/mL) in 25-cm^2^ flasks and cultured until 70–80 % confluency. After 24 h of butein and CORT treatment, cells were fixated with 4 % paraformaldehyde (PFA) (Fujifilm Wako, Japan) and 0.1 % Triton X-100 (Sigma Aldrich, USA). After cells were washed, the γH2AX of the cells was stained using an immunostaining kit including the anti-γH2AX antibody (Dojindo Molecular Technologies, Japan). Nuclei were stained with 5 μg/mL Hoechst 33342 (Dojindo Molecular Technologies, Japan) and observed under a fluorescence microscope (Carl Zeiss, Germany). The percentage of γH2AX positive cells against all cells in the field of view were calculated from 5 fields/flasks randomly selected at 100× magnification using ImageJ (NIH).

### Western blotting analyses

Cells were seeded (2 × 10^5^ cells/mL) in 60 mm dishes and cultured overnight at 37 °C. After 24 h of butein and CORT exposure, the cells were collected with RIPA lysis buffer (ATTO, Japan) containing protease inhibitor (ATTO, Japan), and the harvested lysate was passaged through 26 gauge needles several times to shear genomic DNA, and stored at −80 °C. The protein concentration of each sample was measured with a BCA protein assay kit (Fujifilm Wako, Japan). After addition of loading buffer, equal amounts of cell lysates were separated by SDS-PAGE on 12.5 % gels and electrophoretically transferred to PVDF membranes (ATTO, Japan). Nonspecific binding was blocked with blocking solution (TOYOBO, Japan) at 1 h at room temperature or overnight at 4 °C. The membranes were incubated with primary rabbit monoclonal antibody against cleaved caspase-3 (1:1,000, Cell Signaling Technology, USA) with a reaction solution (TOYOBO, Japan) overnight at 4 °C and washed with TBST three times (3X). The membrane was then incubated with HRP-conjugated secondary antibody (Cell Signaling Technology, USA) diluted 1:10,000 for 1 h at room temperature, then washed with TBST 3X. The immunoreactive bands of the target proteins were detected using an image analyser (Vilber-Lourmat, Germany). GAPDH served as an internal/loading control (Fujifilm Wako, Japan).

### Mitochondrial membrane potential (ΔΨm) assay

To observe mitochondrial damage, we used JC-1 as it shows fluorescence change from green (530 nm) to red (590 nm) depending on the mitochondrial membrane potential (ΔΨm). Cells were seeded in 96-well plates and 25-cm^2^ flasks at a density of 1 × 10^5^ cells/mL. After 24 h of butein and CORT treatment, we measured the fluorescence intensities using the JC-1 detection kit (Dojindo Molecular Technologies, Japan), according to manufacturer instruction.

### Analysis of reactive oxygen species (ROS)

To assess intracellular ROS production, cells were seeded in 96-well plates at a density of 1 × 10^5^ cells/mL and into 60 mm dish (1 × 10^5^ cells/mL). After exposure to butein and CORT, 10 μM Dichloro-dihydro-fluorescein diacetate (DCFH-DA, Sigma Aldrich, USA) was added and cells were incubated for 1 h at 37 °C. Cells were washed twice with HBSS (Fujifilm Wako, Japan) and changes in fluorescence intensity of DCFH-DA were immediately measured using a fluorescence plate reader (Tecan, Switzerland) with excitation at 475 nm and emission at 525 nm. In imaging analysis, nuclei were stained with Hoechst 33342 and observed under a fluorescence microscope (Carl Zeiss, Germany). The ROS generation was evaluated by fluorescence of 2′, 7′-Dichlorodihydrofluorescein (DCF) in combination with N-acetyl-L-cysteine (NAC) (Fujifilm Wako, Japan). NAC is a well-known ROS scavenger, and at final concentration of 50 μM, it was used to identify whether CORT induces or inhibits ROS production.

### Neurite outgrowth assay

Neurite outgrowth was induced in N2A cells by culturing with E-MEM (Fujifilm Wako, Japan) containing 20 μM RA and 2 % FBS for 24 h, according to the method described by Bulfone, et al. (2005). We conducted immunostaining against MAP-2 protein to identify MAP-2 presence in cells during differentiation after RA treatment. Differentiated cells were fixed with 4 % PFA at room temperature for 15 min. Nonspecific binding was blocked for 1 h with 5 % BSA and 0.3 % Triton X-100, and cells were subsequently incubated with primary antibody of MAP-2 (1:200, Cell Signaling Technology, USA) at 4 °C overnight. Cells were then washed and incubated with Alexa Fluor 488 conjugated second antibody (Cell Signaling Technology, USA) for 1 h. The images were captured using a fluorescence microscope (Carl Zeiss, Germany). To evaluate neurite outgrowth for each treatment, 2 × 10^4^ cells/mL were seeded in 60 mm dishes overnight and were exposed to various agents with 20 μM RA and 2 % FBS medium for 24 h. The length of the longest neurite in individual cells from five random fields in each flask, which was seen under a phase-contrast microscope of 200× magnifications, was measured using ImageJ (NIH).

### Statistical evaluation

Experimental values are expressed as the mean ± standard deviation (SD) of four independent experiments. Statistical analysis was performed using one-way ANOVA followed by Tukey's test. A p-value of less than 0.05 was considered significant.

## Results

### Protective effect of butein on CORT-induced cytotoxicity

**Fig. 1:**
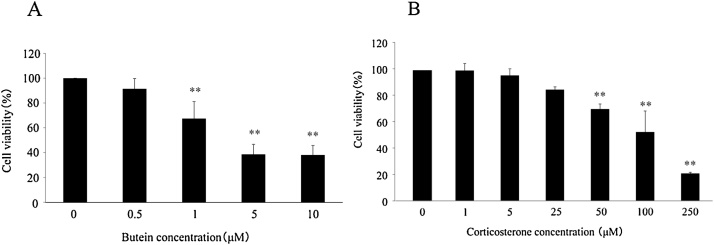
Cell viability after butein and CORT treatment on N2A cells determined by MTT assay. (A) MTT assay results in N2A cells treated with different concentrations of butein for 24 h. Each value is expressed as the mean ± SD of four independent experiments (n = 4) with three technical replicates. (B) MTT assay results in N2A cells treated with increased concentrations of CORT for 24 h. Each value is expressed as the mean ± SD of four independent experiments (n = 4) with three technical replicates. ** p < 0.01 vs control.

**Fig. 2:**
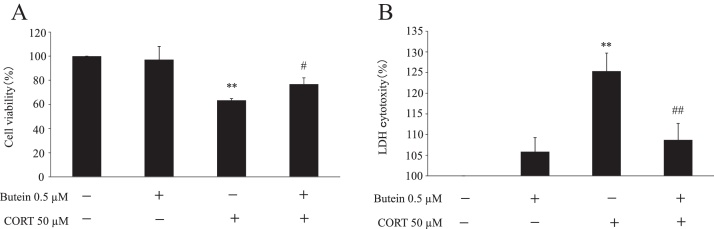
Protective effects of butein on CORT-induced cytotoxicity in N2A cells. (A) MTT assay results in N2A cells treated with 0.5 μM butein for 30 min, then 50 μM CORT for 24 h. Each value is expressed as the mean ± SD of four independent experiments (n = 4) with three technical replicates. (B) LDH assay results in N2A cells treated with 0.5 μM butein for 30 min, then 50 μM CORT for 24 h. Each value is expressed as the mean ± SD of four independent experiments (n = 4) with three technical replicates. ** p < 0.01 vs control; ^#^ p < 0.05 vs CORT group; ^# #^ p < 0.01 vs CORT group.

The viability of N2A cells was determined by an MTT assay to evaluate working concentrations of butein and CORT. N2A cells were cultured with increasing concentrations of butein and CORT for 24 h. Results showed that butein decreased cell viability in a dose-dependent manner (Fig. 1A). At a concentration range of 0.5–5 μM, butein significantly decreased cell viability. When cells were incubated with 50 μM and higher concentrations of CORT, cell viabilities decreased significantly compared with the control group in a concentration-dependent manner (Fig. 1B). Therefore, the resulting 0.5 μM butein did not have a toxic effect on cells, and cells were exposed to 50 μM CORT for 24 h to induce cell damage in subsequent experiments. N2A cells were incubated with 0.5 μM butein for 30 min and a final concentration of 50 μM CORT was added into medium. We cultured cells for 24 h and subsequently performed the MTT and LDH assays to examine the effect of butein (0.5 μM) on CORT (50 μM)-induced cytotoxicity. As shown in Fig. 2A, a significant decrease in cell viability was detected in the CORT groups when compared with the control group. Moreover, a significant difference in cell viability was observed between the CORT only group and butein pre-treatment group. The butein only treated group showed non-significant results in comparison to the group treated with CORT and butein. Results from the LDH assay completely coincided with alterations of cell viability obtained by exposing N2A cells to CORT and butein (Fig. 2B). Although cytotoxicity was indicated marginally in the groups of butein pre-treatment and butein only, cells pretreated with 0.5 μM butein had a significantly lower cytotoxicity than those treated with only CORT. Therefore, these results indicated that butein had a protective effect against CORT-induced cytotoxicity in N2A cells.

### Intracellular mechanism protecting butein against CORT-induced cytotoxicity

**Fig. 3:**
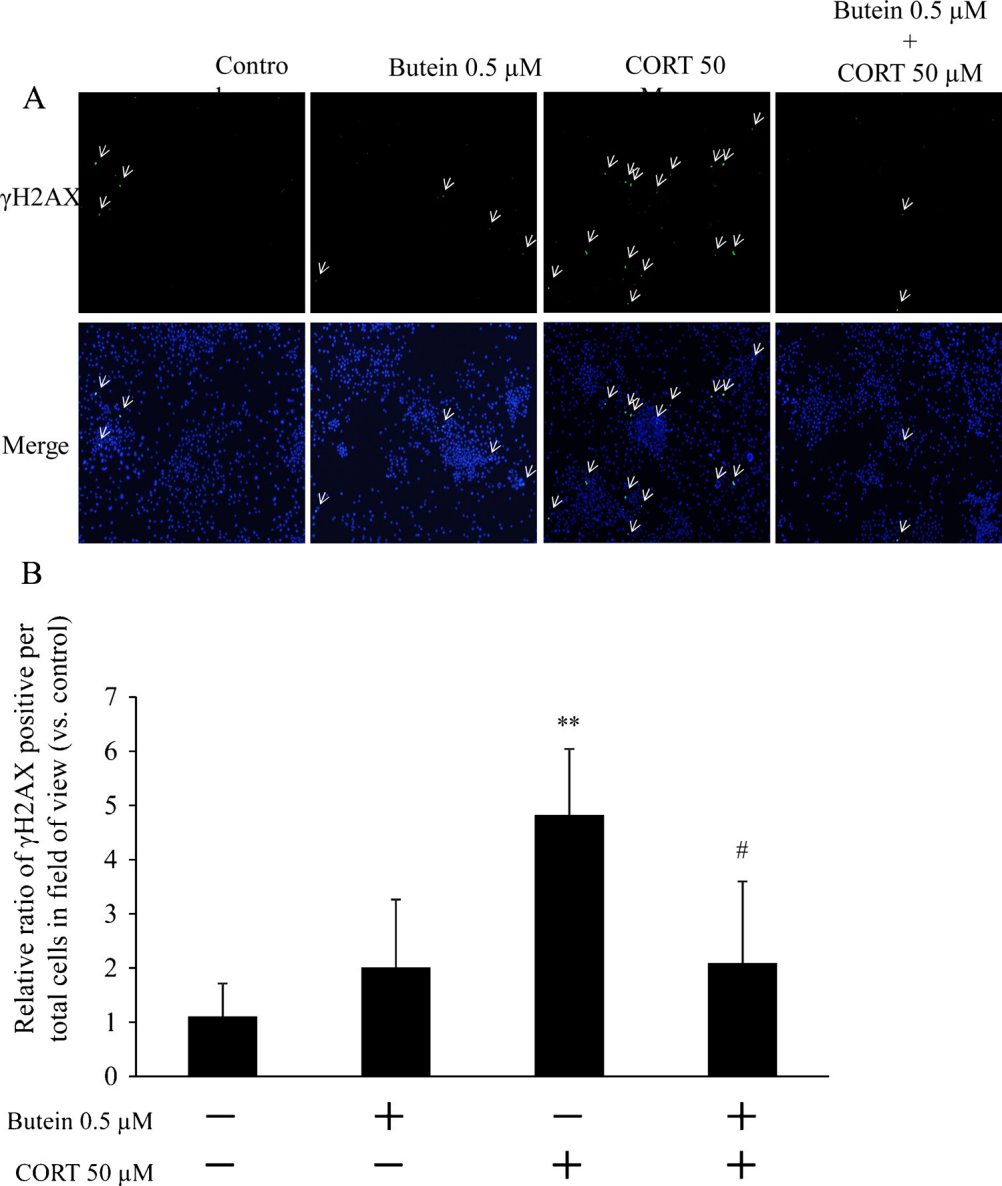
Protective effects of butein pretreatment on CORT-induced DNA damage. (A) Image represented γH2AX (green) staining of cells 100× magnification. The nucleus was stained with Hoechst 33342 (blue). Arrows indicate γH2AX-positive cells. (B) Quantification of γH2AX fluorescence intensity. The percentage of the γH2AX-positive cells against all cells in the observed field were calculated from 5 fields/flasks randomly, and the relative ratio of the γH2AX-positive cells per total cells in the observed field (vs. control) has been expressed as the mean ± SD. ** p < 0.01 vs control; ^#^ p < 0.05 vs CORT group.

We evaluated the protective effect of butein pretreatment on DNA damage by immunostaining γH2AX to detect the damage of DNA levels in CORT-induced cytotoxicity. As per results of imaging analyses (Fig. 3A), cells treated with CORT only remarkably increased green fluorescence in γH2AX staining compared to the control and butein-treated groups. The percentage of γH2AX positive cells treated with CORT increased significantly compared with the control (Fig. 3B). The γH2AX positive cells in butein-pretreated groups were significantly lower than cells treated with CORT only, and there was no significant difference between the butein-pretreated groups and control groups regarding the percentage of γH2AX positive cells.

**Fig. 4:**
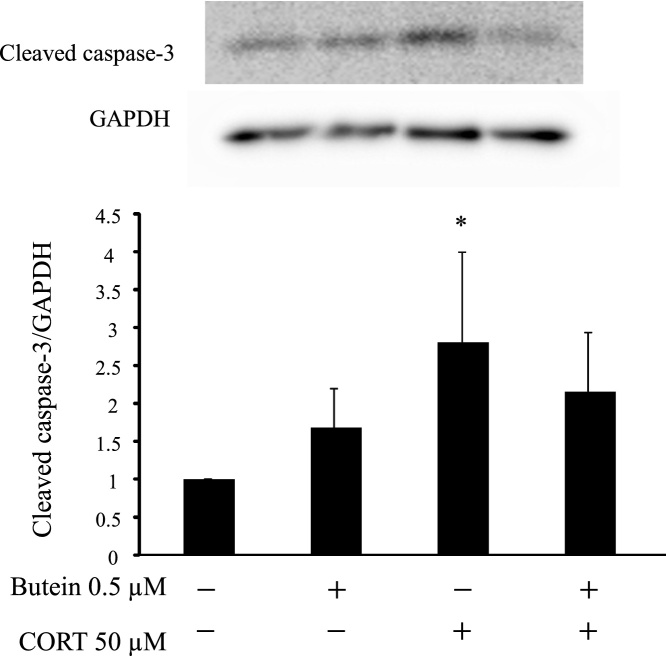
Effect of butein on the change of cleaved caspase-3 in CORT-treated cells. Whole cell lysates were assessed for cleaved caspase-3 expression by western blotting. GAPDH was used as the loading control. Each value is expressed as the mean ± SD of four independent experiments (n = 4). * p < 0.05 vs control.

As changes in caspase-3 cleavage play a key role in the execution of apoptosis, we assessed whether CORT-induced cell death was triggered by caspase activation. As shown in Fig. 4, western blot results showed that the ratio of cleaved caspase-3/GAPDH was increased significantly in CORT-treated cells compared with the control. The levels of cleaved caspase-3 among butein-treated group were lower than those treated with CORT only. Therefore, our results suggests that butein protected CORT-induced apoptosis by regulating caspase signal in N2A cells.

**Fig. 5:**
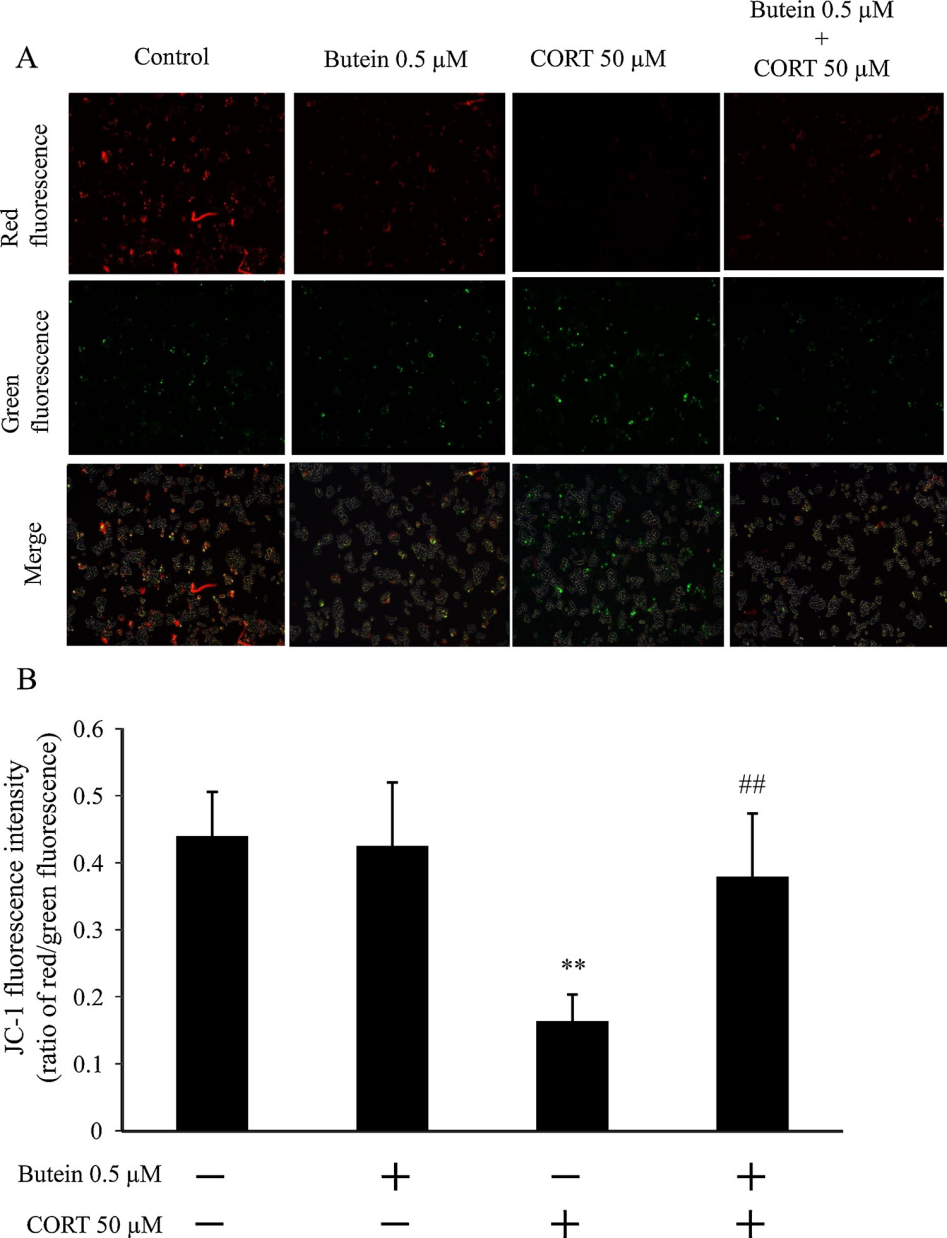
Effect of butein on mitochondrial membrane potential changes (ΔΨm) in the CORT-treated cells. (A) Representative graphical analyses of changes in red and green fluorescence of JC-1 (100× magnification). (B) Quantitative analysis of ratio of red/green fluorescent intensity of JC-1. Each value is expressed as the mean ± SD of four independent experiments (n = 4) with three technical replicates. ** p < 0.01 vs control; ^##^ p < 0.01 vs CORT group.

Mitochondrial dysfunction has been involved in inducing cell apoptosis. We examined the change in ΔΨm by detecting the ratio of the red/green fluorescent intensity of JC-1 staining of cells. As shown in Fig. 5A, cells treated with CORT only reduced the Δψm significantly, compared with the control and cells pretreated with butein. Butein preserved the Δψm loss by treating CORT to N2A cells. Image analysis (Fig. 5B) reflected the result of fluorescence measurements (Fig. 5A). CORT-treated cells remarkably reduced red fluorescence and increased green fluorescence compared with that of control. Although, red fluorescence decreased slightly in the groups of butein pretreatment and butein only, butein pre-treated cells had lower green fluorescence than those treated with CORT only. The results suggest that butein inhibited loss of Δψm in response to CORT treatment and might affect normal Δψm, marginally.

**Fig. 6:**
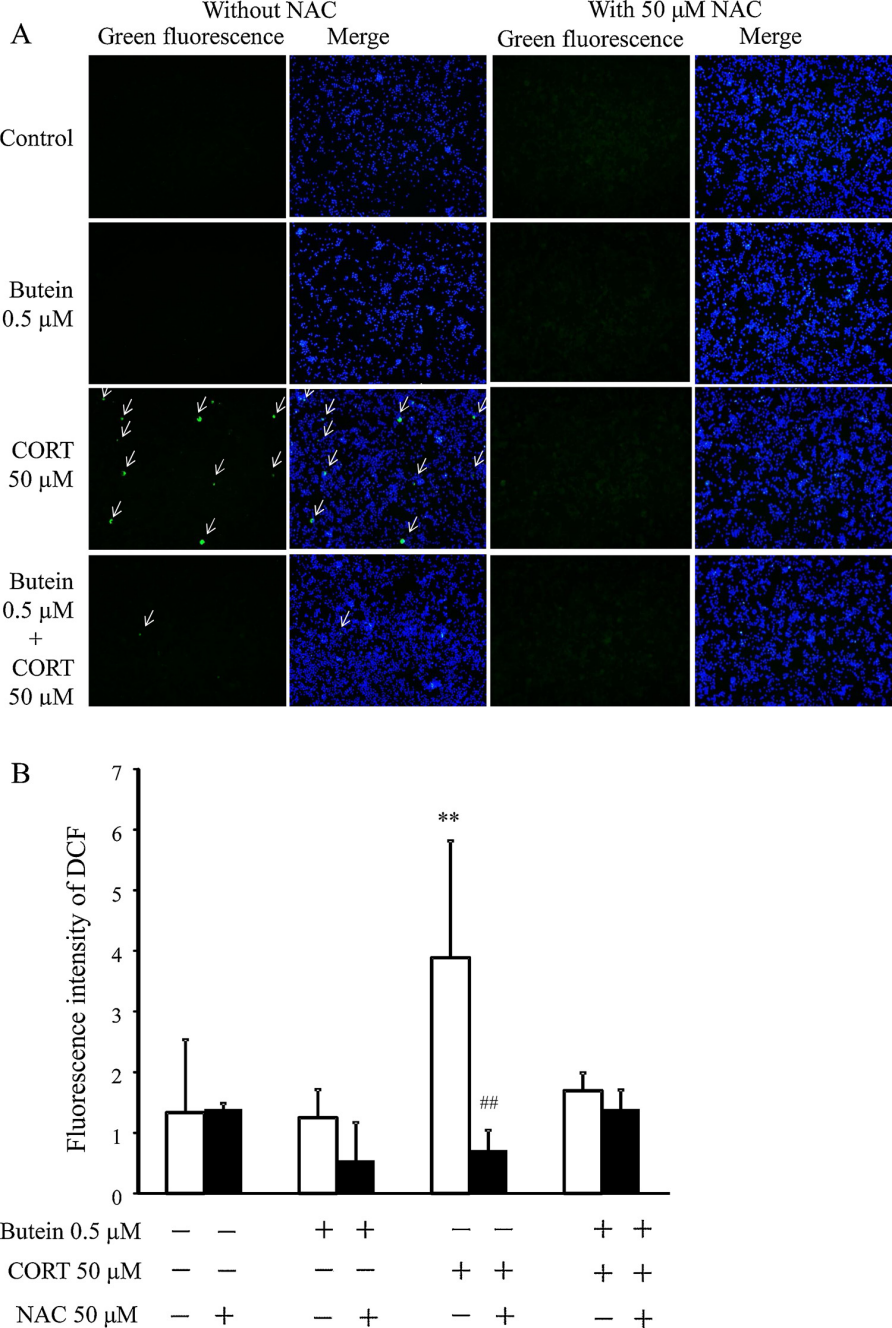
Detection of ROS production. (A) green fluorescence represented the DCF of cells 100× magnification. The nucleus was stained with Hoechst 33342 (blue). Arrows indicate ROS-positive cells. (B) Quantification of fluorescence intensity of DCF: without NAC (white bars) and with 50 μM NAC (black bars). Each value is expressed as the mean ± SD (n = 4) of four independent experiments (n = 4) with three technical replicates. ** p < 0.01 vs CORT group without NAC; ^##^ p < 0.01 vs CORT group without NAC.

The relationship between CORT-treated cell damage, the effect of butein on the damage, and ROS generation was evaluated with the fluorescence of DCF in combination with NAC as a ROS inhibitor. As shown in Fig. 6A, among samples without NAC, cells treated with CORT only showed green fluorescence (DCF), but not the control and butein-treated groups. DCF fluorescence did not show among cells pretreated with NAC. In the quantitative analysis of DCF fluorescence intensity (Fig. 6B) among samples without NAC, CORT-treated group was significantly higher than that of the control. Although no significant differences were found among groups with NAC, CORT-treated cells increased significantly compared to CORT-treated cells with NAC. Therefore, we identified that CORT induces the ROS generation in N2A cells, and butein inhibitorily influenced the ROS generation in CORT-treated cells.

### Effect of butein on neurite growth and differentiation in CORT-treated N2A cells

**Fig. 7:**
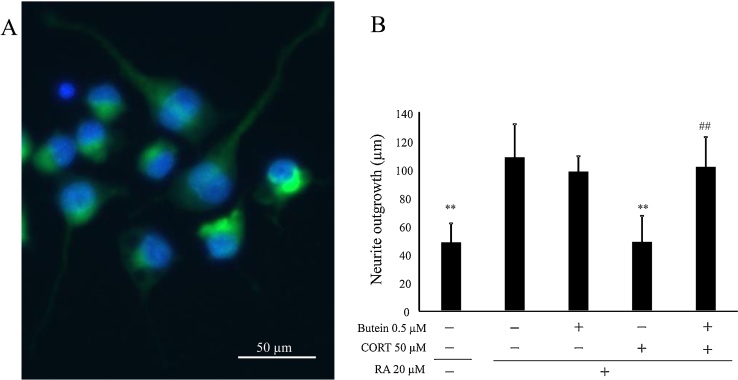
Detection of MAP-2 protein in differentiated N2A cells and protective effects of butein in neurite outgrowth assay of CORT-treated cells. (A) Differentiated N2A cells incubated with 2 % FBS medium, including 20 μM retinoic acid (RA), for 24 h, and immunostained using anti-MAP-2 antibody (green). Cell nuclei were stained with Hoechst 33342 (blue). (B) Average measurements of neurite length were compared among cells with and without RA (positive and negative controls, respectively) for 24 h, exposed to 0.5 μM butein and 50μM CORT with RA for 24 h. Each value is expressed as the mean ± SD of four independent experiments (n = 4). ** p < 0.01 vs positive control; ^##^ p < 0.01 vs CORT group.

We examined the effect of butein on Retinoic Acid (RA)-induced differentiation in N2A cells. Neurites appeared from N2A cells by culturing under low-serum conditions with 20 μM RA for 24 h. As shown in Fig. 7A, cells were stained with the MAP-2 antibody, and the image indicated that MAP-2 protein (green) distributes whole cells from neurite to cell body in differentiated N2A cells. Then, we assessed the effects of butein on neurite outgrowth in cells treated with CORT. Treatment with CORT induced a significant decrease in the average neurite length compared with the control (Fig. 7B). However, the average neurite length of butein-pretreated groups was significantly longer than those of CORT-treated group and did not changed from that of control.

### Effect of high butein concentrations on cultured cells

**Fig. 8:**
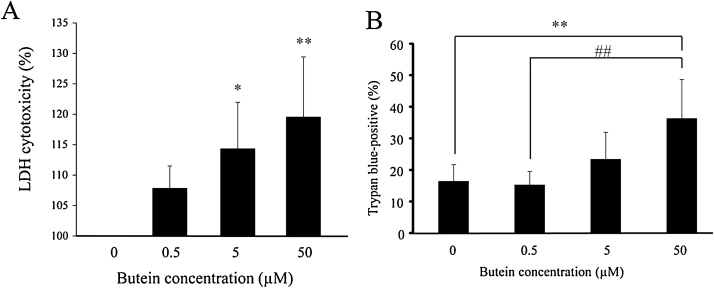
Cytotoxicity of increased butein concentrations on N2A cells. (A) LDH assay results in N2A cells treated with 0.5 μM, 5 μM, and 50 μM of butein for 24 h, compared without butein as control (i.e. 0 μM). Each value is expressed as the mean ± SD of four independent experiments (n = 4) with three technical replicates. (B) Quantification of the percentage of trypan blue-positive cells (dead cell) against all cells in counting field of view cell counting chambers in N2A cells treated with 0.5 μM, 5 μM, and 50 μM of butein for 24 h, compared without butein as control (i.e. 0 μM). Each value is expressed as the mean ± SD of four independent experiments (n = 4). * p < 0.05 vs control; ** p < 0.01 vs control; ^##^ p < 0.01 vs 50 μM butein.

**Fig. 9:**
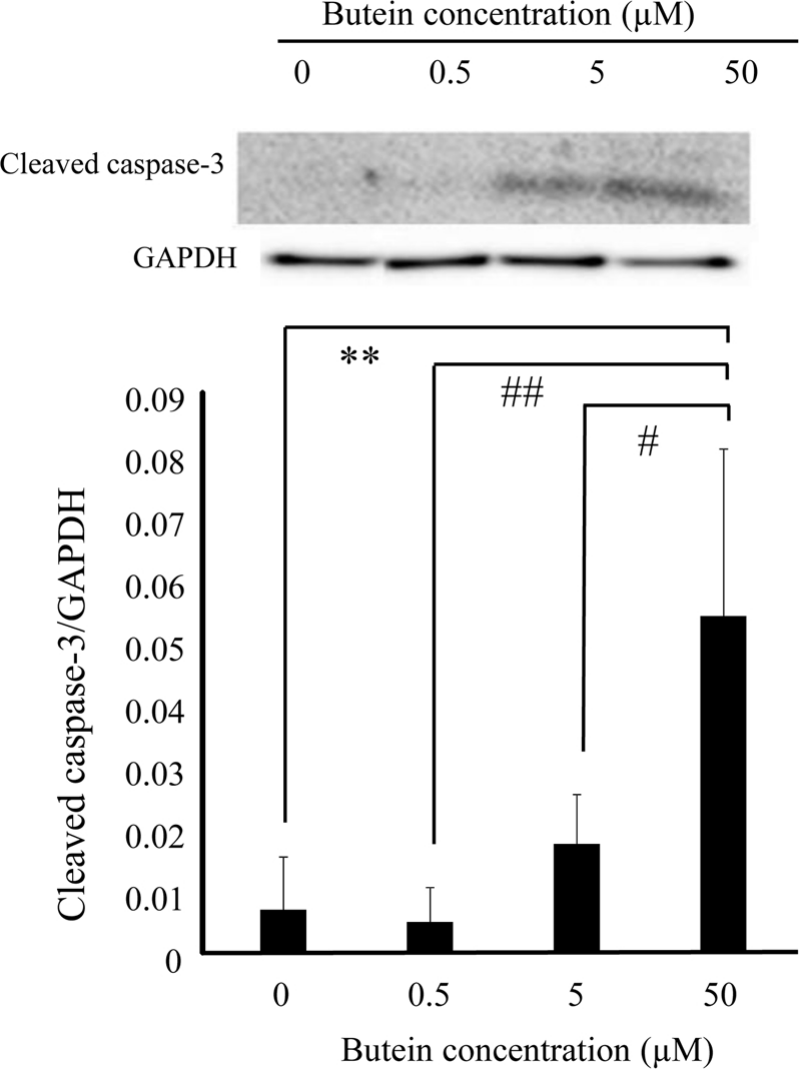
Effect of increased concentrations of butein on the change of cleaved caspase-3 in N2A cells. Whole cell lysates were assessed for cleaved caspase-3 expression by western blotting. GAPDH was used as the loading control. Each value is expressed as the mean ± SD Each value is expressed as the mean ± SD of four independent experiments (n = 4). ** p < 0.01 vs control; ^#^ p < 0.05 vs 50 μM butein; ^##^ p < 0.01 vs 50 μM butein.

We performed the LDH assay to investigate any cytotoxic effects resulting from high concentrations of butein for a period of 24 h. To further confirm the results obtained from the LDH assay, we counted the number of cells after staining with trypan blue. Our result demonstrated that butein induced LDH leakage in a dose-dependent manner (Fig. 8A). Concerning cells treated with 5–50 μM butein, the cytotoxicity was increased significantly compared with the control. Additionally, the percentage of trypan blue positive cells treated with 50 μM butein was significantly higher than those treated with 0.5 μM butein and the control (Fig. 8B). Although a significant difference was not observed, the percentage of trypan blue positive cells treated with 5 μM butein increased compared with the 0.5 μM butein-treated cells. The contribution of cleaved caspase-3 was confirmed in the process of cell death due to high butein concentrations after 24 h of treatment. As shown in Fig. 9, the western blot analysis showed that the ratio of cleaved caspase-3/GAPDH increased significantly in 50 μM butein-treated cells compared with other treatments, as reflected in the results of the cytotoxic assay from LDH leakage and trypan blue staining. The significant cytotoxicity increase in 50 μM butein-treated cells may be attributed to apoptosis facilitation by activating caspase-3. Therefore, these results suggest that butein cytotoxicity against N2A cells may be enhanced by using relatively higher butein concentrations than 0.5 μM.

**Fig. 10:**
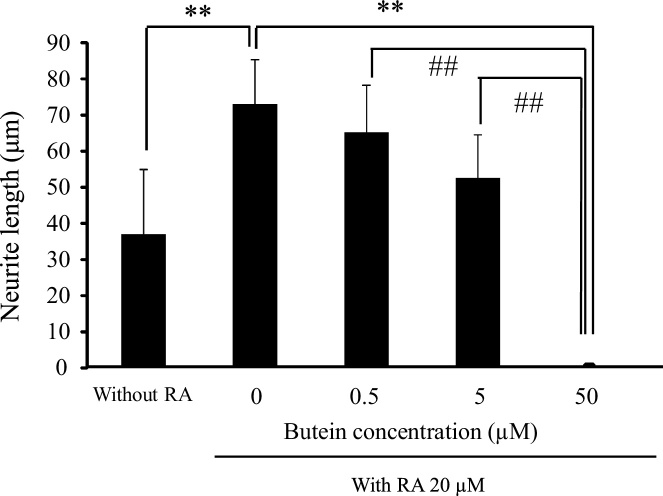
Effect of increased concentrations of butein on the change of average neurite length in N2A cells as compared among cells without RA (negative control) and with 0 μM (positive control), 0.5 μM, 5 μM, and 50 μM of butein with RA for 24 h. Each value is expressed as the mean ± SD of four independent experiments (n = 4). ** p < 0.01 vs positive control; ^##^ p < 0.01 vs 50 μM butein.

We examined the effect of different concentrations of butein on RA-induced differentiation in N2A cells. Assay results showed a butein dose-dependent inhibition of neurite outgrowth (Fig. 10). Cells treated with 50 μM butein significantly decreased in average neurite length compared with the control and other butein concentrations. In cells untreated with RA, a small amount of neurite could be confirmed, however, cells treated with 50 μM butein fully lost all neurites. As butein concentrations increased, cell number conversely decreased. Consequently, our results suggest that cells treated with 50 μM butein might undergo atrophic cell dysfunction owed by apoptotic damage.

## Discussion

The present study demonstrated that butein protects N2A cells from CORT-induced cytotoxicity. The protective effects were indicated as the results of pre-treatment of N2A cells with 0.5 μM butein prior to 50 μM CORT exposure. Cells treated with 50 μM CORT significantly decreased cell viability and induced cell death. Previous studies suggest that CORT induces apoptosis via mitochondrial dysfunction, caspase-3 activation, and ROS production. This apoptosis may be attributed to DNA damage on the basis of γH2AX detection as a trigger of intracellular ROS increase by CORT. Interestingly, butein pretreatment significantly inhibited CORT-induced cell cytotoxicity, ROS production, LDH leakage, caspase-3 activation, loss of Δψm, and DNA damage.

Zhang et al. (2012) reported that exposure of primary cultured hypothalamic neurons to CORT for 24 h resulted in increased LDH release, intracellular levels of ROS, caspase-3 activity, and decreased mitochondrial function. However, pretreatment with the flavonol glycoside icariin prior to CORT exposure was identified to prevent CORT-treated cell apoptosis by significantly inhibiting these intracellular changes. According to the report by Liu et al. (2011), the protective effects of icariin on CORT-induced cell apoptosis have also been demonstrated in hippocampal neurons of primary cultured rat, which were consistent with results from primary cultured hypothalamic cells. In an additional report the protective effects of butein on ROS-related apoptosis in cultured neuron cells, Lee and Jeong (2016) demonstrated that butein effectively prevents glutamate-induced oxidative damage in mouse hippocampal HT22 cells and primary mouse hippocampal neurons. Glutamate also produced ROS in HT22 cells and induced cell apoptosis via DNA fragmentation, increase of caspase-3 activation, and decrease of Bcl-2 and Bcl-xL expression. Butein dose-dependence suppressed ROS production and the alteration of intracellular molecules while undergoing apoptosis. From the experiments using NAC to confirm ROS production, we suggested that butein suppressed ROS generation. The antioxidant activity of butein may exert blockage of ROS generation to relate to the loss of Δψm, activation of caspase cascade, and DNA damage, against CORT-induced apoptosis in N2A cells.

Moreover, we determined whether CORT inhibits neurite outgrowth in N2A cells and investigated the protective effect of butein against CORT-induced neurodevelopmental toxicity. Our results show that CORT significantly suppressed RA-induced neurite outgrowth in N2A cells; however, butein significantly protected the negative effect of CORT. Previous studies show that the modulation of ROS levels influences multiple aspects of neuronal differentiation and brain function. Munnamalai and Suter (2009) demonstrated that decreased levels of ROS by a free radical scavenger reduced the F-actin content in neurons and ultimately resulted in disassembly of the actin cytoskeleton. When neurons were cultured overnight in conditions of reduced free radicals, growth cone formation and neurite outgrowth were severely impaired. Physiological levels of ROS are suggested to be critical for maintaining a dynamic F-actin cytoskeleton and controlling neurite outgrowth, whereas Tsatmali et al. (2006) reported that increases in ROS levels are transient *in vivo* during neuronal development because excessive ROS plays a neurotoxic role during neuron differentiation. Furukawa et al. (2019) reported that their synthesized carbazole derivative have the ability to protect N2A cells from hydrogen peroxide-induced cell death and induce neurite outgrowth through activation of PI3K/Akt signalling in N2A cells. Therefore, we speculate that butein might inhibit CORT-induced ROS generation and prevent inhibition of RA-induced neurite outgrowth via an intracellular signalling modification similar to that resulting from butein protectivity under CORT-induced apoptosis.

Our study demonstrated that butein may induce more apoptotic cytotoxicity in N2A cells in higher concentrations than at 0.5 μM. This cytotoxicity significantly affected RA-induced differentiation in the N2A cells at 50 μM butein in particular. We used a concentration of 0.5 μM butein in various assays because the viability of cells treated with 0.5 μM butein had not decreased significantly (Fig. 1A). Chen et al. (2012) reported that butein induced apoptosis in N2A cells in a dose-dependent manner through decreased Bcl-2/Bax ratio and increased cleavage forms of caspase-3 and PARP. This apoptosis was caused by ROS production at higher butein concentrations in accordance to our results. With regards to other neuronal cells, according to the reported MTT assay in HT22 cells (Lee and Jeong. 2016), 10 μM butein have showed no cytotoxic effects while a higher concentration of 20 μM slightly reduced cell viability. Although the reported relationship between cell cytotoxicity and butein dose-dependence has been naturally different due to the use of various cell lines and experimental methods, butein might induce apoptosis in N2A cells in relatively higher butein concentrations under consistent experimental settings.

Chronic stress affects structural changes and neuronal damage in the hippocampus and decreases BDNF in the dentate gyrus (Smith et al., 1995). Chronic administration of several antidepressant drugs significantly increased BDNF mRNA in the hippocampus, and could promote neuronal survival and protect neurons from the damaging effects of stress (Nibuya et al., 1995). BDNF expression in brain is known to increase in subjects treated with antidepressants compared with antidepressant-untreated subjects (Chen et al., 2001), and BDNF levels were significantly lower in patients of major depression (Karege et al., 2002). CREB and BDNF play an important role in neurogenesis and synaptic plasticity in vital areas such as the hippocampus and the cortex for learning, memory, and cognition (Hashimoto et al., 2004). Increased BDNF expression by CREB phosphorylation results in increased secretion of BDNF, which acts via TrkB receptors and activates the MAPK signalling pathway. MAPK signalling phosphorylates CREB and regulates cellular survival by increasing the expression of the anti-apoptotic protein Bcl-2. Cho et al. (2013) reported that the effects of butein on CREB phosphorylation and BDNF expression in the hippocampus of scopolamine-induced amnesic mice was determined, and western blotting analysis showed no effect of CREB phosphorylation and slightly increased the BDNF expression. Although *in vitro* studies showcased the potent neuroprotective effects of butein, no apparent correlation between the *in vitro* neuroprotective effects and *in vivo* enhancing effects of butein were found. While the pharmacokinetic properties of butein are unclear, the bioavailability of flavonoids *in vivo* is low, generally due to limited absorption. In humans, peak plasma concentrations of polyphenols in the range of 0.1–10 μmol/L have been found to be obtained after oral consumption, thereafter, the flavonoid in the blood is metabolized extensively and excreted rapidly (Kroon et al., 2004). Most circulating flavonoids are flavonoid metabolites, some of which have lower antioxidant activity than the parental flavonoid (Lotito et al., 2011). The actual contribution of flavonoids to biological effects *in vivo* might be limited compared with *in vitro* studies using nonmetabolites of flavonoids.

In conclusion, we identified that 0.5 μM butein can prevent the 50 μM CORT-induced apoptosis in N2A cells via protective effects on ROS generation, mitochondrial dysfunction, and caspase-3 activation, and may also rescue the negative effect of 50 μM CORT on RA-induced differentiation in N2A cells. Furthermore, the butein dose-dependent cytotoxicity in N2A cells was demonstrated. The biological activities of butein on neural cells and the brain are exhibited by the modulation of various intracellular signalling pathways, such as the MAPK and the PI3K/Akt pathways. For future studies, we need to clarify the molecular interaction of relevant signalling pathways underlying the protective effect of butein on CORT-induced apoptosis in N2A cells and to validate the results using primary neuronal cells instead of N2A cells (i.e. neuroblastoma). The present study provides preliminary results demonstrating the beneficial role and potential of butein in neuroprotection.

## Authors’ contributions

M.O. designed the study, carried out the experiments, performed statistical analyses, and drafted the manuscript. Y.S., S.T., and T.M. participated in the experiments, and performed statistical analyses. N.M., Y.I., and T.D. participated in the design of the study. All authors read and approved the final manuscript.

## Conflicts of interest

All authors declare having no competing interests.
